# Brazilian Consensus on the Application of Thermal Ablation for Treatment of Thyroid Nodules: A Task Force Statement by the Brazilian Society of Interventional Radiology and Endovascular Surgery (SOBRICE), Brazilian Society of Head and Neck Surgery (SBCCP), and Brazilian Society of Endocrinology and Metabolism (SBEM)

**DOI:** 10.20945/2359-4292-2023-0263

**Published:** 2024-06-24

**Authors:** Gustavo Philippi de los Santos, Marco Aurélio Vamondes Kulcsar, Fabio de Aquino Capelli, Jose Higino Steck, Klecius Leite Fernandes, Cleo Otaviano Mesa, Joaquim Mauricio da Motta-Leal-Filho, Rafael Selbach Scheffel, Fernanda Vaisman, Guilherme Lopes Pinheiro Martins, Denis Szejnfeld, Mauricio Kauark Amoedo, Marcos Roberto de Menezes, Antonio Rahal, Leandro Luongo Matos

**Affiliations:** 1 Hospital Universitário Universidade Federal de Santa Catarina Florianópolis SC Brasil Hospital Universitário da Universidade Federal de Santa Catarina, Florianópolis, SC, Brasil; 2 Sociedade Brasileira de Cirurgia de Cabeça e Pescoço São Paulo SP Brasil Sociedade Brasileira de Cirurgia de Cabeça e Pescoço, São Paulo, SP, Brasil; 3 Faculdade Israelita de Ciências da Saúde Albert Einstein São Paulo SP Brasil Faculdade Israelita de Ciências da Saúde Albert Einstein,São Paulo, SP, Brasil; 4 Hospital das Clínicas Faculdade de Medicina Universidade de São Paulo São Paulo SP Brasil Serviço de Cirurgia de Cabeça e Pescoço, Instituto do Câncer do Estado de São Paulo, Hospital das Clínicas, Faculdade de Medicina, Universidade de São Paulo, São Paulo, SP, Brasil; 5 Hospital das Clínicas Faculdade de Medicina Universidade de São Paulo São Paulo SP Brasil Instituto do Câncer do Estado de São Paulo, Hospital das Clínicas, Faculdade de Medicina, Universidade de São Paulo, São Paulo, SP, Brasil; 6 Divisão de Otorrinolaringologia Universidade Estadual de Campinas Campinas SP Brasil Divisão de Otorrinolaringologia, Universidade Estadual de Campinas, Campinas, SP, Brasil; 7 Hospital Mario Gatti Campinas SP Brasil Hospital Mario Gatti, Campinas, SP, Brasil; 8 Universidade Federal da Paraíba João Pessoa PB Brasil Universidade Federal da Paraíba, João Pessoa, PB, Brasil; 9 Serviço de Endocrinologia e Metabologia Hospital de Clínicas Universidade Federal do Paraná Curitiba PR Brasil Serviço de Endocrinologia e Metabologia, Hospital de Clínicas, Universidade Federal do Paraná, Curitiba, PR, Brasil; 10 Faculdade de Medicina Pontifícia Universidade Católica do Paraná Curitiba PR Brasil Faculdade de Medicina, Pontifícia Universidade Católica do Paraná, Curitiba, PR, Brasil; 11 Departamento de Radiologia Instituto do Câncer do Estado de São Paulo São Paulo SP Brasil Departamento de Radiologia, Instituto do Câncer do Estado de São Paulo, São Paulo, SP, Brasil; 12 Hospital das Clínicas Faculdade de Medicina Universidade de São Paulo São Paulo SP Brasil Instituto do Coração, Hospital das Clínicas, Faculdade de Medicina, Universidade de São Paulo, São Paulo, SP, Brasil; 13 Serviço de Tireoide Hospital de Clínicas de Porto Alegre Porto Alegre RS Brasil Serviço de Tireoide, Hospital de Clínicas de Porto Alegre, Porto Alegre, RS, Brasil; 14 Departamento de Farmacologia Universidade Federal do Rio Grande do Sul Porto Alegre RS Brasil Departamento de Farmacologia, Universidade Federal do Rio Grande do Sul, Porto Alegre, RS, Brasil; 15 Instituto Nacional do Câncer Rio de Janeiro RJ Brasil Instituto Nacional do Câncer (INCA), Rio de Janeiro, RJ, Brasil; 16 Universidade Federal do Rio de Janeiro Rio de Janeiro RJ Brasil Universidade Federal do Rio de Janeiro, Rio de Janeiro, RJ, Brasil; 17 Hospital Sírio-Libanês São Paulo SP Brasil Hospital Sírio-Libanês, São Paulo, SP, Brasil; 18 Hospital Samaritano São Paulo SP Brasil Hospital Samaritano, São Paulo, SP, Brasil; 19 Departamento de Radiologia Intervencionista Universidade Federal de São Paulo São Paulo SP Brasil Departamento de Radiologia Intervencionista, Universidade Federal de São Paulo, São Paulo, SP, Brasil; 20 Radioclínica Salvador BA Brasil Radioclínica, Salvador, BA, Brasil; 21 Hospital Santa Izabel Salvador BA Brasil Hospital Santa Izabel, Salvador, BA, Brasil; 22 Santa Casa da Bahia Salvador BA Brasil Santa Casa da Bahia, Salvador, BA, Brasil; 23 Hospital da Bahia Salvador BA Brasil Hospital da Bahia, Salvador, BA, Brasil; 24 Departamento de Oncologia Sociedade Brasileira de Radiologia Intervencionista e Cirurgia Endovascular São Paulo SP Brasil Departamento de Oncologia, Sociedade Brasileira de Radiologia Intervencionista e Cirurgia Endovascular, São Paulo, SP, Brasil; 25 Hospital das Clínicas Faculdade de Medicina Universidade de São Paulo São Paulo SP Brasil Centro de Intervenção Guiada por Imagem, Instituto do Câncer do Estado de São Paulo, Hospital das Clínicas, Faculdade de Medicina, Universidade de São Paulo, São Paulo, SP, Brasil; 26 Centro de Intervenção Guiada por Imagem Hospital Sírio-Libanês São Paulo SP Brasil Centro de Intervenção Guiada por Imagem, Hospital Sírio-Libanês, São Paulo, SP, Brasil; 27 Área do Núcleo de Imagem e de Intervenção em Tireoide Hospital Israelita Albert Einstein São Paulo SP Brasil Área do Núcleo de Imagem e de Intervenção em Tireoide, Hospital Israelita Albert Einstein, São Paulo, SP, Brasil; 28 Sociedade Paulista de Radiologia São Paulo SP Brasil Radiologia Intervencionista, Sociedade Paulista de Radiologia, São Paulo, SP, Brasil

**Keywords:** Thyroid, treatment, ablation techniques, radiofrequency ablation, interventional ultrasound

## Abstract

There is increasing interest in ultrasound-guided ablation treatments for thyroid diseases, including benign and malignant ones. Surgeons, radiologists, and endocrinologists carry out these treatments, and various organizations within these specialties have recently released multiple international consensus statements and clinical practice standards. The aim of the present consensus statement is to provide guidance, cohesion, and standardization of best practices for thermal ablation procedures of thyroid nodules. The statement includes the indications for these procedures, preprocedural evaluations, technical aspects of the procedures, posttreatment care, follow-up, complications, and training recommendations. This document was written by a panel of specialists from the Brazilian Society of Interventional Radiology and Endovascular Surgery (SOBRICE), the Brazilian Society of Head and Neck Surgery (SBCCP), and the Brazilian Society of Endocrinology and Metabolism (SBEM). The statement does not aim to provide criteria for assessing the capability of specialists to perform the procedure. Instead, it aims to promote the standardization of best practices to reduce potential adverse outcomes. Additionally, it strives to enhance the delivery of high-quality care and the widespread adoption of these technologies on a national level. The recommendations collectively serve as a guidebook for applying best practices in thyroid ablation.

## INTRODUCTION

There is increasing interest in ultrasound (US)-guided ablation treatments for thyroid diseases, both benign and malignant ones. Surgeons, radiologists, and endocrinologists carry out these treatments, and various organizations within these specialties have recently released multiple international consensus statements and clinical practice standards on this topic (1-8). Given the multidisciplinary and international collaboration involved, it is crucial to identify the optimal clinical practices for US-guided ablation treatments specifically in Brazil. An essential aspect of this process is the emphasis on standardizing the indications, terminology, reports, and techniques associated with these procedures (9,10).

The aim of this consensus statement is to provide guidance, cohesion, and standardization of best practices for conducting thermal ablation procedures on thyroid nodules. It is worth noting that the primary focus of this guideline is not the well-established practice of chemical ablation using ethanol or other sclerosing agents, for which there is extensive scientific evidence (1).

As the utilization of ablation technologies continues to grow across diverse clinical settings, it has become imperative to establish criteria for selecting patients for these procedures. These standards enable the evaluation of efficacy and encourage the proper application of these developing technologies. The use of ablation technologies in benign and malignant diseases that have gathered sufficient data and drawn significant attention in the medical literature will be examined in this publication. Nodules that cause cosmetic issues or compression symptoms and autonomously functioning thyroid nodules are examples of benign disorders. Malignant diseases include small, low-risk primary thyroid tumors classified as microcarcinomas or papillary thyroid microcarcinomas (PTMCs), as well as recurrent cervical (lymph node) metastases from thyroid cancer. This consensus statement focuses on evidence-based practices and ethically acceptable procedures conducted outside a research environment (2,6-8,11-14).

It is essential to emphasize that specific training is necessary for performing US and US-guided procedures and is a prerequisite for safely applying US-guided thermal ablation technology (15). This statement does not aim to provide criteria for assessing the capability of specialists to perform the procedure. Instead, it aims to promote the standardization of best practices to reduce potential adverse outcomes. Additionally, it strives to enhance the delivery of high-quality care and the widespread adoption of these technologies on a national level. The recommendations collectively serve as a guidebook for applying best practices in thyroid ablation.

## ABLATIVE METHODS

Ablation methods can be performed chemically or thermally.

### Chemical ablation or sclerotherapy

Ablation therapy with ethanol or another sclerosing agent is a procedure in which a sclerosing substance, commonly absolute alcohol, is injected into the target organ to induce tissue sclerosis. It is the preferred treatment for thyroid nodules that are predominantly cystic or mixed with a predominant (>50%) cystic component confirmed to be benign. Several retrospective and prospective studies have demonstrated a 50%-98% reduction in nodule volume and improvement in local symptoms after ethanol injection (1,3,4). These positive outcomes have been sustained for up to 5 years after treatment. Multiple interventions may be required in multilocular lesions or nodules larger than 10 mL (1,16-20).

To enhance treatment efficacy and reduce the need for additional interventions, alcoholization can be combined with thermal ablation in large and mixed nodules. The implementation of these two procedures, in combination or separately, depends on the patient’s requirements and the nodule’s characteristics (21-24).

### Thermal ablation

All thermal ablation techniques involve the destruction of tissue by subjecting it to extremely high temperatures. The primary mechanism of cellular death in thermal ablation is coagulation necrosis. Temperatures below 40 °C are associated with reversible cellular damage without long-term effects (11,25,26), while temperatures in the 50-60 °C range cause irreversible injury, with the damage increasing more rapidly as the temperature rises. The objective of ablation techniques is to maintain temperatures within the range of 50-60 °C for 4 to 6 minutes. When the temperature exceeds 60 °C, protein denaturation and cell membrane disruption lead to immediate tissue necrosis, which forms the basis for the “moving-shot” technique (25,26). Temperatures above 100-110 °C result in tissue vaporization and carbonization, generating gas around the electrode. This gas formation can insulate the tissue and hinder the diffusion of heat, thereby limiting the effectiveness of ablation (27,28). Thermal ablation techniques are categorized based on the method used to create this temperature differential.

### Radiofrequency ablation

Radiofrequency ablation (RFA) is a technique that eliminates the target tissue by creating heat through friction and conduction (27) and employing a high-frequency alternating current that ranges from 200 kHz to 1,200 kHz (25,29). The RFA waves cause frictional heat when they pass through an electrode, agitating tissue ions as they try to follow the alternating current’s changing directions. As a result, the tissue a few millimeters away from the electrode and surrounding the needle’s active area becomes hotter. The cells suffer immediate and permanent thermal damage because of heat transfer to the ablated area, which affects tissues further from the electrode (25,28,29).

The moving-shot technique is used in thyroid treatment to reduce the effects of heat conduction, with thermal energy predominantly produced from frictional heat (30). In monopolar mode, the patient joins the generator, electrode needle, and two grounding electrodes in a closed circuit that disperses the energy. The current in bipolar mode is contained in the needle tip and does not penetrate the patient. Both the tissue temperature and the length of heating determine how much tissue damage is caused by RFA (26,28).

The “heat-sink” effect, which refers to tissue cooling brought on by perfusion from nearby blood arteries, as well as tissue heterogeneity (such as calcification, fibrosis, or fluid present), may impede the conduction of electricity and heat and lessen the efficiency of RFA (28).

In benign thyroid nodules, RFA has proven to be the most efficient US-guided ablation approach for solid, mixed, and nonfunctioning sponge-like lesions (31-34). Compared with surgery, RFA treatment of benign nodules is associated with a lower risk of complications, higher quality of life, and better thyroid function preservation (35-41).

Limited data exist regarding the efficacy of RFA therapy for hyperthyroidism associated with hyperfunctioning nodules, and long-term follow-up is lacking. However, available studies demonstrate the potential indication and safety of RFA in these nodules (6,42-47).

The effectiveness of RFA in eradicating recurrent or residual thyroid cancer ranges from 68% to 93%, according to several studies (12,48-52). Surgical reintervention and RFA have comparable percentages of recurrence-free survival and posttreatment voice change. In the context of recurrent thyroid cancer, a study has found that 91% of the tumors remained radiographically undetectable after 5 or more years, although long-term follow-up data is scant (52,53). Even in cases in which comprehensive disease therapy is unfeasible, palliative RFA may alleviate symptoms or halt the spread of local malignancy (52).

A comprehensive cohort study with 414 patients with unifocal PTMC treated with RFA showed a complete US response in 88% of the tumors and an average volume decrease of 98.8% after 42 months in the context of primary neoplasia, with 3.6% of the patients experiencing locoregional progression (54). Comparable levels of effectiveness have been found in several studies assessing the use of RFA in low-risk PTMC without signs of metastatic illness (54-58). It is important to note that the use of RFA in the treatment of primary thyroid carcinoma is still developing.

### Laser thermal ablation

Thyroid nodules have been treated with laser thermal ablation (LTA), an alternative technique for delivering thermal energy (3). In LTA, an optical cable is introduced into the target tissue, directing a concentrated beam of light energy (59). Heat transmission to the thyroid results in local tissue effects caused by photon dispersion (28). Energy absorption has unique properties that may be tuned to each tumor and patient since it is nonlinear and temperature-dependent (60). Presently, numerous laser sources, optical fibers, and wavelengths are available. The LTA technique most commonly employed for thyroid LTA involves either a neodymium:yttrium aluminum garnet (Nd:YAG) laser or a diode laser with an emission wavelength of 1,064 nm, coupled with planning and modeling software (28). Notably, LTA offers less total energy than conventional thermal ablation methods, improving safety and control in crucial locations (3).

Supported by both experimental and clinical data, the use of LTA for the treatment of benign thyroid nodules was developed in 2000 (61), and the efficacy of this technique has been shown in numerous studies (62-66). Additionally, LTA has been researched as a potential treatment for autonomously functioning thyroid nodules. Patients with smaller autonomous thyroid nodules treated with LTA tend to have favorable outcomes similar to those of RFA (67).

The effectiveness of LTA in the management of PTMC has yet to be thoroughly investigated. A retrospective study analyzing LTA treatment of PTMCs has shown that 94% of the ablated lesions disappeared completely on US examination, which is comparable to the result achieved with RFA (68). A systematic review and meta-analysis of 1,284 lesions treated with RFA, LTA, or microwave ablation (MWA) for PTMC found no significant differences in volume reduction or major complications between the three techniques (69).

### Microwave ablation

The MWA method induces the oscillation of polarized ions, specifically water molecules, by applying an electromagnetic field with wavelengths of 0.03-30 cm and frequencies of 900-2,500 MHz (70). The friction this oscillation produces raises the temperature in the immediate area (71). Unlike RFA, which depends on an electric current, MWA relies on an electromagnetic field and operates without requiring electrical conduction. As a result, the tissue carbonization and heat dissipation (heat-sink effect) in MWA have a lower impact on thermal propagation (72). The current is typically propagated using an antenna, and numerous antennas may be active simultaneously. When these antennas are phased, the heating increases exponentially. As a result, MWA enables the transmission of greater thermal energy in a shorter time, raising the tissue’s final temperature (71). For larger tumors, this faster treatment is very beneficial. Because it is effective in treating large tumors and tissues (*e.g.*, liver, lungs, and bone) that interfere with the propagation of electrical current, MWA has grown in popularity (73,74).

Some factors may pose difficulties for MWA treatment in the thyroid. The compact anatomical structure of the thyroid in the cervical region and the fast heating and lower heat-sink effect from MWA may explain some of the difficulties noted in some initial studies evaluating this technique (3,75-77). These issues, however, have not been reported in more recent studies (75,78,79).

Few studies have been performed on the use of MWA for thyroid nodules, and there is still a need for long-term follow-up data. Theoretically, MWA has an advantage over other thermal ablation techniques because of its larger ablation zone and lower heat-sink effect (80). However, there may be an increased risk of discomfort and complications with MWA (81). Nevertheless, several studies (75,81-84) have shown that MWA is effective in treating thyroid nodules.

### High-intensity focused ultrasound

High-intensity focused US (HIFU) is a noninvasive procedure that utilizes high-frequency sound waves to target lesions or focal areas. While low-frequency sound waves are nondestructive and frequently employed in physical therapy to boost the body’s natural response to injury, high-frequency sound waves deliver enough energy to cause coagulative necrosis through thermal and mechanical harm (85). The thermal effect is produced by transferring the energy from strong tissue vibration into frictional heat. High temperatures are produced inside the selected target area, and cell death occurs when the temperature hits 55-60 °C. This temperature range causes microbubbles to develop and tissue-internalized water to evaporate. The expansion and contraction of these microbubbles cause mechanical damage and cell hemorrhage in neighboring cells through a process known as cavitation (86). A key component of HIFU is the ability to accurately deliver energy to a small area while protecting the tissues around the area. This is accomplished by concentrating high-intensity waves from several external sources at one point, preventing excessive energy buildup along the propagation path (87).

Data from prospective studies evaluating HIFU treatment of thyroid nodules are currently limited. Two systematic reviews on HIFU for nonfunctioning benign nodules reported reductions in nodular volume of 45%-70% over 3-24 months of follow-up (88). In a retrospective multicenter European trial, a single HIFU session was associated with a nodular volume reduction of 30%-35% (89). In thyroid nodules treated with HIFU, the initial size of the nodule and the degree of volume reduction have an inverse relationship, as observed with other thermal ablation procedures (90,91). Although a retrospective study comparing thyroid lobectomy to HIFU for symptomatic benign nodules was constrained by selection bias due to larger nodules in the surgical group, it still showed a volume reduction of 51.7% at 6 months after nodular ablation in the HIFU group, along with shorter hospital stays, lower costs, greater symptom improvement, and fewer effects on voice (92). Data on HIFU treatment of autonomously functioning nodules are limited, but a comparison from a single institution indicated significantly better scintigraphic response and resolution of hyperthyroidism in patients treated with radioiodine than in those treated with HIFU (93). The available evidence for HIFU is generally of low level and predominantly based on retrospective or single-center studies.

## Conclusions

Retrospective studies, randomized prospective trials, and sizable multicenter studies with extended follow-up provide strong evidence for the efficacy of thermal ablations in the treatment of benign and nonfunctioning thyroid diseases (94). However, there are currently no large-scale studies with long-term follow-up addressing the best uses of these approaches in autonomously functioning or malignant nodules.

In minimally invasive procedures for thyroid nodules, the long-term outcomes are influenced by the characteristics of the nodule, its location, and the extent of its internal vascularization (94). As a result, when making decisions, it is important to consider the various learning curves, technical requirements, and equipment complexity linked to each technology. To avoid complications, physicians should be cautious while performing thermal ablation. Over time, the results should match those of studies on this topic.

## INDICATIONS OF THERMAL ABLATION IN THE BENIGN CONTEXT

Patients with large benign thyroid nodules who experience compressive or cosmetic symptoms should consider treatment with RFA (2-5). When contemplating treatment with US-guided thermal ablation, physicians should consider several factors associated with the patient and the disease. Size, determined through US measurement, is the most objective criterion for determining the need for the procedure in benign thyroid nodules. However, variables such as body mass index, neck circumference, and location of the nodule within the thyroid may cause patients with nodules of similar sizes to experience different levels of discomfort. For instance, it is difficult to estimate the size requirements for ablation of isthmic nodules because they frequently cause more obvious cosmetic complaints sooner in their development (2-5).

According to some recommendations, ablation should begin in nodules with a minimum diameter of 20-30 mm and continued growth visible on US (2,3,6). Compressive symptoms and aesthetic issues are considered valid clinical reasons for intervention because it is difficult to define specific size requirements. A visual analog scale ranging from 0 (no symptoms) to 10 (highest symptoms) can be used to score symptoms. On the other hand, physicians can assess the cosmetic score on a scale of 1-4 (2). The evaluation process may also be assisted by quality-of-life surveys and forms related to thyroid nodules (95,96).

Before performing ablation of a benign thyroid nodule, it is generally recommended to confirm a benign cytological diagnosis through two US-guided fine-needle aspirations (FNAs) or core needle biopsies (CNBs), as outlined in previous guidelines (2,3,6,97). In cases in which the US characteristics highly suggest benignity, such as spongiform aspect, purely cystic nodules, or functional nodules, a second FNA may not be necessary but is still recommended. It is important to avoid ablation of nodules with suspicious US findings, even with two benign FNAs, to prevent overlooking or delaying the treatment of a malignant lesion (2-5).

Cross-sectional imaging should be used to assess the presence of retrosternal extension thoroughly; if the extension is considerable, the patient is ineligible for ablation (2-5). Functional thyroid nodules may be treated with thermal ablation (42,98). With success rates ranging from 24% to 72% (43-45,98), it is important to remember that the resolution of hyperthyroidism following ablation is less predictable with thermal ablation than with RAI or surgical treatment. A recent meta-analysis has shown TSH normalization after 12 months in 71.2% of the cases of autonomous thyroid nodules treated with thermal ablation (99). Patients undergoing ablation for autonomous nodules have lower risks of procedure-related problems and hypothyroidism than those undergoing surgery. However, thyroid function normalization occurs in fewer patients (46). Since efficacy is linked to a > 80% reduction in nodule volume, ablation is best suited for patients with small nodules (≤3.5 cm) in whom radioiodine or surgery is contraindicated (3,46). Ablation is less effective in toxic multinodular goiter and is not advised in Graves’ disease; thus, scintigraphy is recommended to determine the presence of an autonomously functioning nodule (3,100). For both functional and nonfunctional nodules, TSH levels should be recorded before ablation, and free T4 levels should be assessed whenever the TSH level is outside the normal range.

## INDICATIONS FOR THE USE OF THERMAL ABLATION IN THE CONTEXT OF MALIGNANCY AND FOLLICULAR NEOPLASIA

In patients with recurrent thyroid malignancies in the thyroid bed and cervical lymph nodes who are at high surgical risk or choose not to undergo surgery, RFA can be used as a curative or palliative treatment (7,8). The objective of treating malignant thyroid nodules with RFA is to create a thermal ablation zone that completely encloses the nodule, leaving a safety margin of at least 2 mm in the surrounding healthy thyroid tissue. Inadequate ablation at the nodule’s margins may leave viable thyroid cells behind and increase the likelihood of late recurrence (101-103).

Surgery is currently the standard treatment for primary thyroid cancer; as a result, the indications for ablation in primary thyroid cancers have not been fully established or extensively studied yet (7,8). Various forms of ablation, such as RFA, LTA, and MWA, are being investigated in the treatment of patients with PTMC. However, these treatment modalities have been primarily studied in small case series and controlled environments. Additionally, active surveillance is increasingly recommended as the initial approach for cases of PTMC with favorable patient and tumor characteristics and with access to appropriate monitoring by health care professionals (104,105). Therefore, thermal ablations are currently reserved as a second-line treatment for patients with primary PTMC who are unable to undergo surgery or are not suitable candidates for active surveillance.

Several medical societies propose active surveillance as the initial strategy for patients with PTMC who are considered ideal or appropriate candidates based on tumor and patient characteristics and health care coverage (105). Surgical intervention is recommended for patients who are deemed unsuitable for active surveillance. It remains unclear in which scenarios thermal ablation would be indicated for these tumors, as no randomized studies have compared this modality versus active surveillance or demonstrated its superiority over tumor observation alone (7,8).

After ablation, a thorough evaluation is required to look for recent lymph node metastases or the probable recurrence of papillary thyroid cancer (PTC) in other thyroid gland regions. When there is a suspicion of recurrence, computed tomography (CT) is an important adjunct to US in the identification of recent lymph node metastases (7). According to earlier research, the remaining lesion after ablation undergoes degenerative anatomical and pathological changes. As a result, only lesions that grow or remain stable in size are advised for CNB or FNA (56,106).

In recent years, numerous major studies have been conducted to evaluate the application of RFA for malignant nodules (34,54,58,107,108). Initially, these studies focused on assessing the outcomes of thermal ablative therapy in malignant nodules in patients with high surgical risk, where conventional surgical treatment could lead to increased morbidity and mortality, or in patients who decline surgical treatment and active surveillance. Similarly, many studies have examined the treatment of patients with tumor recurrence in the thyroid bed or lateral lymph node metastases, where surgery carries a high risk of morbidity and mortality (12,48,109-115).

The safety and effectiveness of thermal ablation in PTC have been examined in 40 trials that were analyzed in a recent review by Ou and cols. (116). In total, 5,268 low-risk PTC nodules were submitted to thermal ablation in 5,074 adult patients (mean age 45 ± 4 years) with small PTCs (mean maximum size 6 ± 3 mm). The studies in this review had a mean follow-up duration of 3.3 years and were mostly conducted in Chinese institutions. More than 50% of the ablations were carried out utilizing RFA, approximately 25% with MWA, and 12.5% with LTA. While 73% of the studies limited ablation to a single thyroid cancer, 27% included patients who underwent ablation of multiple nodules within the same lobe. The authors concluded that the ablation techniques are well established for low-risk T1aN0M0 PTMCs and can achieve similar efficacy with fewer complications compared with surgery.

Currently, the study with the longest follow-up duration is a retrospective cohort study by Kim and cols. (107) that reported on the safety and efficacy of percutaneous laser ablation in 90 patients followed up for 10 years.

A cohort study conducted by Cho and cols. (58) investigated 74 patients who underwent RFA for PTMC treatment, with a total of 84 nodules treated with ablation. After ablation, the patients were evaluated at 6 and 12 months and annually thereafter for 6 years (72 months). All patients tolerated the ablation treatment well. Complete disappearance of the tumors was achieved in 98.8% of patients within the first 2 years and in 100% after 6 years. Additional ablations were performed in 13 out of the 84 thyroid nodules (15%), with an average of 1.2 ablation sessions. Four new cancers (metachronous) emerged in 3 patients, and they were also treated with RFA, resulting in complete disappearance. No local tumor progression, lymph node metastasis, or distant metastasis occurred during the 72-month follow-up period. None of the patients required rescue surgery, and no late complications related to the procedures were reported (117).

In a literature review published by Min and cols. (14), the results for the three ablation techniques (RFA, LTA, and MWA) were satisfactory, with tumor volume reduction reaching or exceeding 99% over a follow-up period of 6-64.2 months. Ablative therapies showed a significantly lower incidence of complications than conventional surgical treatments. Objective criteria, such as blood loss, length of hospital stay, and total hospital costs, were also significantly lower in ablative therapies, leading to improved quality of life for the patients (117).

Another study by Ntelis & Linos (118), also conducted in 2020, reviewed 33 studies from the MEDLINE and SCOPUS databases, focusing on RFA as a treatment for low-risk PTCs. The study included 1,289 patients and 1,422 tumors. The average volumetric reduction ranged from 47.8% to 100%, with most studies showing reductions between 98.5% and 100%. Complete disappearance of tumors was observed in 33.7% to 100% of the studies, with longer follow-up studies reporting reduction rates between 56% and 100%. Tumor progression and signs of recurrence occurred in 0-4.5% of the cases. Complications were reported in 45 patients (3.2%), with mild to moderate pain and cervical discomfort being the most common. No life-threatening complications were reported. The study concluded that RFA is an effective and safe alternative for the treatment of low-risk PTCs in patients at high surgical risk or in those who refuse surgery (118).

A study by Song and cols. (119), published in 2021, aimed to assess the risks and benefits of ablation for PTMCs located in the isthmus. The retrospective study included two groups of patients: in one group, the tumors were treated with RFA, and in the other, with total thyroidectomy. At 12 and 18 months, the rates of disappearance of the ablation zone in the RFA group were 90.4% and 100%, respectively. Compared with the RFA group, variables such as procedure/surgery time, blood loss, hospital stay, and overall treatment expenses were all greater in the total thyroidectomy group. The RFA group’s final score in the thyroid cancer-specific quality-of-life questionnaire (THYCA-QOL) was much lower than that of the total thyroidectomy group. According to the study’s findings, RFA treatment of isthmic PTMC produced results similar to those of total thyroidectomy but with fewer risks and lower costs (119).

A meta-analysis by Shen and cols. (120), which included 658 patients followed up for an average of 3.5 years, compared thermal ablation with partial thyroidectomy. Compared with immediate surgery, thermal ablation was associated with significantly fewer complications, a shorter postoperative hospital stay, and lower perioperative spending. Differences in recurrence rates and disease-free survival rates between the two groups were not significant. In 2020, a review article by Rangel and cols. (121) on RFA ablation of benign and malignant thyroid nodules found results similar to those of the meta-analysis. Table 1 provides the current recommendations for the appropriate selection of patients for percutaneous thermal ablation in low-risk PTMC (116,122,123).

**Table 1:** Appropriate patient selection for thermal ablation of low-risk papillary microcarcinoma, according to Tuttle and cols. (122)

	Nodule Characteristics	Patient Characteristics	Medical Team Characteristics
Appropriate Case	Nodule ≤ 1 cm confined to the thyroid, surrounded by ≥ 2 mm of normal thyroid tissue, no signs of extrathyroidal extension, absence of cervical metastasis	Has a strong desire to preserve thyroid function, is at high risk or is ineligible for surgery, is unwilling to accept active surveillance, prefers a minimally invasive approach, has a strong desire to avoid surgery, is willing to accept an investigational therapeutic approach, understands the possibility of new cancer foci or cervical metastases during follow-up in the remaining thyroid gland	Experienced and trained in thermal ablation techniques, availability of specialists for evaluating and treating potential complications, proper collection of prospective data, physician with previous experience in ultrasound and ultrasound-guided fine-needle aspiration biopsy, access to appropriate thermal ablation equipment
Inappropriate Case	Nodule located adjacent to the recurrent laryngeal nerve, near the trachea and major vessels, evidence of capsular invasion, aggressive cytology, molecular examination indicating aggressiveness, N1 or M1 disease	Has a history of neck irradiation, desires surgical intervention, prefers active surveillance, is reluctant to accept a new therapeutic approach	Reliable ultrasound not available, lack of experience with thermal ablation techniques

Patients with advanced thyroid cancer may benefit from ablation as a palliative care option. Both RFA and LTA have been considered as palliative treatments for anaplastic or advanced medullary thyroid carcinoma (8,113). While other investigators were unable to see any therapeutic effects of thermal ablation on advanced anaplastic or medullary malignancies (113), some have reported improvements in compressive symptoms in patients with advanced anaplastic cancer receiving LTA.

Because a previously treated area is being treated in the case of recurrent thyroid cancer, the overall complication rate is higher (11%) (103,124,125). There have been several reported complications, including major ones (permanent hypothyroidism, nodular rupture, and injuries to the recurrent laryngeal nerve, cervical sympathetic ganglion, brachial plexus, and spinal accessory nerve) and minor ones (hematoma, vomiting, skin burns, transient thyrotoxicosis, lidocaine toxicity, hypertension, and pain) (126,127). However, no potentially fatal complications have occurred, and the rate of long-term complications is 0.21% (13,125,128).

Follicular adenomas are one of the most frequent thyroid nodules, and follicular carcinomas account for 10%-20% of all malignant thyroid lesions. Capsular, vascular, or extrathyroidal tissue invasion, as well as lymph node or distant metastasis, are parameters used to distinguish between follicular cancer and follicular adenoma (129). Surgery is the preferred method of treatment for follicular neoplasia based on the results of FNA or CNB (8). Because there is currently insufficient information regarding the advantages of thermal ablation treatment for follicular neoplasms (2,5), ablation is not advised in these cases. Obtaining a definitive pathological confirmation to rule out malignancy before surgery is challenging in follicular neoplasia (129). In this type of neoplasia, a tumor size (>2 cm) is a predictor of malignancy (129,130). These tumors frequently appear as thyroiditis or nodular goiter in surgical pathology, with FNA showing a high false-positive rate (22.2%-35%) (129). Consequently, there is an increasing demand for conservative medical treatment for patients at high surgical risk or ineligible for surgery. There is little research on RFA for follicular neoplasms, although several articles have reported on the safety and effectiveness of this procedure for treating benign thyroid nodules and even recurrent thyroid cancer (130,131). However, a recent study with a follow-up of 5 years has found RFA to be an efficient and secure approach for treating follicular neoplasms smaller than 2 cm, with no recurrences or metastatic lesions observed during the follow-up period (130). In another study, the authors opposed the use of RFA as a first-line treatment for follicular neoplasms, as two of the six lesions in this trial that were greater than 2 cm in size and were classified as Bethesda III regressed after RFA but were ultimately determined to be minimally invasive follicular carcinoma and follicular neoplasm of undetermined malignant activity (132). In Bethesda III lesions, the authors hypothesized that RFA may increase the risk of residual cancer and delay surgery in situations of malignancy (132). In other tumor types, tumor development in different organs has been reported as a result of incomplete thermal ablation therapy (133-136). Therefore, current recommendations do not support the use of thermal ablation for the management of indeterminate thyroid nodules (2,8,132).

## PREPROCEDURAL EVALUATION

When evaluating a patient for US-guided thermal ablation, several factors related both to the disease itself and the patient should be considered (137). The most objective criterion for assessing eligibility for the procedure in a benign thyroid nodule is the size of the nodule on US evaluation. The severity of symptoms may differ greatly among patients with nodules of comparable size, as it may be influenced by different factors such as body mass index, neck circumference, and position of the nodule within the thyroid (137). For instance, nodules located in the isthmus typically produce more visibility and early aesthetic issues. It can be challenging to define the ideal size requirements for ablation; however, earlier proposals recommended a minimum diameter of 20-30 mm on US in nodules that continue to grow (2,3,6). However, the presence of compressive symptoms and cosmetic issues are thought to be legitimate clinical indications for intervention, given the difficulties in establishing objective size criteria. The degree of symptoms and aesthetic impact can be evaluated by visual symptom rating and cosmetic scoring by the physician (2). Tools such as the Short Form Health Survey (SF-36) or the Thyroid-Related Patient-Reported Outcome (ThyPRO) can be used to assess quality-of-life metrics (95,96).

During the perioperative period, US evaluation plays a crucial role in identifying anatomical landmarks for safe ablation (137). Particular attention should be given to the tracheoesophageal groove, known as the “danger triangle,” where the recurrent laryngeal nerve (RLN) is not visible but remains vulnerable to thermal injury. Transfixion of the jugular veins should be avoided to minimize the risk of hematoma. Injury to the vagus nerve, located between the internal jugular vein and carotid artery in the carotid sheath, and the sympathetic chain, which courses deep and laterally to the carotid artery, should also be avoided (137). In lateral cervical treatments, the physician should be aware of the potential locations of the accessory nerve, brachial plexus, and other neural structures (137).

Before the ablation, voice evaluation is necessary and should be documented during the physical examination (138,139). The patient should be asked about any voice abnormalities or changes in tone, intensity, or quality. To ascertain the existence and severity of voice impairment, additional examination using validated measures may be considered (140,141). Evaluation of vocal cord mobility requires a laryngeal examination, for which the gold standard is transnasal or transoral endoscopic examination. In patients with favorable anatomy, laryngeal US can also be used to evaluate vocal cord mobility (142). This examination becomes particularly important if the patient has undergone previous thyroid or neck surgery. Voice evaluation alone is not sufficient to predict objective vocal cord function and does not replace laryngeal examination in higher-risk populations (143).

Evaluation of the patient’s overall health and medical history is crucial for the patient’s preparation and counseling and for choosing the appropriate procedure site (117,144). The physician should ask the patient about other medical conditions, use of medications, previous surgeries, and pregnancy status. Significant cardiopulmonary comorbidities and the presence of pacemakers, implantable defibrillators, or arrhythmias may require treatment under continuous monitoring. Sedation, instead of local anesthesia alone, should be considered for patients with extreme anxiety, chronic opioid use, fibromyalgia, cognitive impairments, or low tolerance to procedures under local anesthesia (117,144).

Managing anticoagulation in patients undergoing thermal ablation can be complex, particularly with the use of novel oral anticoagulants (145). The best approaches should consider the procedure’s potential for bleeding, the anticoagulant’s pharmacokinetics, and the patient’s potential for thromboembolic events. Aspirin and clopidogrel should generally be stopped 7 days prior to the surgery, whereas warfarin should be stopped 5 days beforehand. Three days before surgery, users of more recent oral anticoagulants, such as dabigatran, rivaroxaban, apixaban, and edoxaban, should interrupt their use. All anticoagulant medications can be safely resumed 24 hours after surgery (145).

Before any invasive procedure, comprehensive guidance should be provided to the patients and their companions regarding indications, risks, and potential complications (137). Informed consent should be obtained, and the consent form should include information about the gradual reduction in the size of ablated thyroid nodules over time, the expected number of treatment sessions, the possibility of nodule regrowth and need for additional treatment, potential symptoms during and after ablation, complications from RFA, disclosure of thyroid surgery history and medications, and the potential need for further observation or hospitalization depending on the patient’s postprocedural condition.

## INDICATIONS AND RECOMMENDATIONS FOR PREPROCEDURAL EVALUATIONS

**Recommendation 1. Ultrasound-guided ablation procedures can serve as a primary alternative to surgery in patients experiencing compressive and/or aesthetic symptoms due to benign thyroid nodules.**

**Recommendation 2. Thermal ablation procedures, while not as effective as surgery or radioiodine therapy in normalizing thyroid function, can be a safe therapeutic option for patients with hyperfunctioning (autonomous/Plummer) thyroid nodules who are unable to undergo first-line therapies due to contraindications.**

**Recommendation 3a. Ultrasound-guided ablation procedures may be considered as a second-line treatment for patients with primary thyroid carcinoma up to 1 cm in size and a favorable location when surgery or active surveillance is not feasible.**

**Recommendation 3b. Ultrasound-guided ablation procedures may be considered for patients with recurrent metastatic papillary carcinoma with a favorable location who are not suitable for or decline surgery.**

**Recommendation 3c. Preprocedural biochemical and imaging evaluations are important to determine whether the goal of treatment for recurrent papillary thyroid carcinoma is curative or palliative.**

**Recommendation 3d. Chemical (ethanol) ablation is recommended for cystic or predominantly cystic nodules with more than 50% liquid content causing symptoms or cosmetic concerns but is not recommended for solid or predominantly solid nodules.**

**Recommendation 4a. Subjective and, if possible, objective assessment of voice should be performed in all patients before ultrasound-guided ablation procedures.**

**Recommendation 4b. Patients with vocal alteration or significant previous surgical history should undergo laryngeal evaluation and assessment of vocal cord mobility prior to the ablative procedure.**

**Recommendation 4c. Vocal fold mobility should be assessed before ablation on the contralateral side following ipsilateral ablation.**

**Recommendation 5a. Comprehensive patient evaluation, including history, physical examination, imaging, and biochemical tests, should be performed prior to ultrasound-guided ablation procedures. The use of a checklist and symptom scale can aid in the evaluation process.**

**Recommendation 5b. Before proceeding with ultrasound-guided ablation procedures, the expected outcomes and potential risks should be discussed with the patient.**

**Recommendation 5c. Informed consent should be obtained from the patient before the ablation procedure.**

## TECHNICAL NOTES

### Radiofrequency ablation

While the technical aspects described in the following passage specifically relate to RFA, they generally apply to other ablation techniques, such as LTA, HIFU, and MWA. The choice of ablation type depends on the physician’s preference and the patient’s comfort and safety (2).

Prior to the procedure, if sedation is administered, the patient should avoid heavy meals or fasting and have a companion present for assistance. The patient is placed in a supine position with slight neck extension, while the physician stands at the side or head of the table. It is important to note that if the physician is at the head, the US images will appear inverted on the screen.

In RFA, grounding pads are placed on both anterior thighs, distal to the neck, after confirming the absence of metal in the body (*e.g.*, jewelry, piercings, hearing aids, clothes hooks, or wires) to prevent conduction-related injuries. The patient’s eyes may be covered to prevent inadvertent injuries. The neck skin is cleansed with an aseptic solution and covered with a sterile drape. In some cases, a mild sedative may be administered to help the patient remain relaxed during the procedure (2,3).

Vital signs should be monitored both before and after the procedure, and additional blood pressure, heart rate, and pulse oximetry monitoring may be considered, particularly if anesthesia is being given. Depending on the established standards for sedative treatments in adults, different levels of monitoring may be necessary (144). It is advisable to have emergency supplies and oxygen close at hand in the unlikely event of cardiac arrhythmia during the procedure (97,144).

The procedure of RFA for thyroid nodules consists of three main technical components: local anesthesia, transisthmic approach, and moving-shot technique (2,15,51,146). To ensure patient comfort during RFA, adequate local anesthesia (*e.g.*, 1% lidocaine) should be injected at the skin puncture site and around the thyroid capsule to prevent pain (99). Both the skin and the thyroid capsule have sensory nerves, but the gland itself does not. In accordance with the US evaluation obtained before the procedure and the required trajectory to reach the target nodule, the local anesthetic is injected at the anticipated site(s) on the anterior portion of the neck where the RFA electrode will be implanted. Lidocaine is also injected around the thyroid to create a clear separation between the gland and the overlying sternothyroid muscle, which appears as an anechoic band on US evaluation. The total amount of lidocaine administered is determined based on the patient’s weight and renal function (147,148). Local anesthetic infiltration also serves the purpose of hydrodissection, which helps separate the thyroid from nearby vulnerable structures and creates a fluid barrier for heat dissipation. Cooled 5% glucose solution may be used for hydrodissection as it does not transmit electricity and creates a thermal barrier surrounding the target organ (149,150).

Complications associated with ablation, such as voice change and ptosis, can be effectively monitored during local anesthesia, but their detection may be delayed during general anesthesia (151). Patients should report any new neck pain because it could be a sign that the thyroid capsule is heating up and spreading its heat beyond the thyroid gland. In such cases, the treatment should be paused, and additional local anesthetic may be administered or the RFA electrode repositioned. Inadvertent damage brought on by electrode displacement during speaking, which should be avoided when the electrode tip is active, can be avoided using the patient’s nonverbal communication.

To treat a nodule in the right or left thyroid lobe, the transisthmic method entails inserting the electrode across the isthmus from medial to lateral (152,153). This method enables ongoing observation of the relationship between the electrode, the target nodule, and the area around the RLN, which is located within the “dangerous triangle.” The target nodule and the electrode insertion site are separated by a healthy isthmus parenchyma, which prevents heated ablative fluid from seeping into the perithyroidal region and potentially causing pain or thermal injury. The transisthmic technique further minimizes the danger of damage by guaranteeing secure electrode positioning even if the patient speaks, drinks, or coughs. A more paramedian approach, however, might be necessary for true isthmic nodules (25,27). The moving-shot technique is suggested for treating thyroid nodules as opposed to the fixed strategy utilized for ablating tumors in other organs, such as the liver. This is because thyroid nodules are frequently elliptical, bulky, and exophytic, which poses challenges in obtaining consistent therapy with a set approach and can lead to undertreatment or overtreatment of the nodule’s perimeter and surrounding tissue. A better approach is to separate the thyroid nodule into several smaller ablation units and treat each one separately. As the electrode tip is withdrawn, the process moves from treating the deepest area of the nodule to treating the most superficial section. Ablation should be suspended when the electrode moves forward during repositioning (27,154).

The power settings are determined by the operator, starting conservatively at approximately 40-50 W and potentially increasing up to 70 W, depending on the patient’s tolerance, to maximize ablation efficiency. The heat generated and the ablation area can extend approximately 3-5 mm beyond the electrode tip, and electrode advancement should consider the size of the active tip, power level, and proximity to vital structures (23,25,27). Real-time US should be used to track electrode tip placement continuously during treatment. Depending on the size of the nodule and the length of the ablation, the operator can anticipate applying 15-50 kJ of energy to a benign thyroid nodule. Techniques for vascular ablation have been developed to reduce the recurrence of the nodule or regrowth after treatment.

Hypervascular nodules with a significant feeding artery may be treated with nutrient artery ablation (155). This procedure lowers the possibility of bleeding, which could obstruct thermal conduction, as well as edema and the heat-sink effect in hypervascular tumors. Finding the nodule’s primary arterial supply with Doppler US is helpful. The marginal drainage veins that contribute to the heat-sink effect and obstruct (up to 75%) complete ablation of the nodule edge are the focus of marginal vein ablation. The anterior portion of thyroid nodules, where the marginal veins are, has a significant safety margin for ablation, making thyroid nodules a good candidate for this type of treatment. Additionally, the marginal veins can help prevent the recurrence of the nodules (155,156). A wider margin is required for the treatment of functional nodules, microcarcinomas, and recurrent lymph node disease to eliminate the nodule completely. The moving-shot technique should cover the surrounding soft tissue of the recurrent tumors in cases of thyroid cancer (152,157,158). Before ablation, the tumor’s position in relation to important neck structures, such as the RLN, esophagus, and trachea, should be assessed (154). Hydrodissection becomes essential to separate the tumor from these important structures in these situations (149,150,158). Treatment of small primary lesions and/or recurrent malignancies is successful and safer with an electrode with a small active tip (0.5 cm) (159).

To obtain the best outcomes with thyroid RFA, the right tools must be used. Electrodes made especially for thyroid RFA have different tip sizes (*i.e.*, 5, 7, and 10 mm). The dimensions of the nodule being treated and the level of precision required will determine the tip size. Smaller electrodes must be used on nodules that are close to vital structures or in high-risk locations. A 10 mm electrode might be considered for larger nodules (>4 cm) to speed up the ablation process. Smaller nodules requiring more treatment area control are only treated with the 5 mm electrode (159,160). Multiple modifications of the ablation tip can now be made using a single device thanks to the development of electrodes with adjustable active tips (160). Although bipolar electrodes are also available, monopolar electrodes are currently more frequently used in thyroid RFA. More concentrated energy is provided by bipolar electrodes, where the current only passes between the electrodes at the device’s tip. In patients who are pregnant or who have implanted cardiac electrical devices, the use of bipolar electrodes may be safer (159). In Brazil, bipolar electrodes are accessible. Additional developments have been made to increase the safety of ablative treatments for thyroid nodules. When the ablation target immediately abuts a crucial structure, unidirectional ablation electrodes, which are spaced to provide a narrower and more focused ablation zone, may be considered (159). To facilitate monitoring of the electrode tip, virtual needle tracking technologies have also been created and are constantly being enhanced. While they are more advantageous for fixed ablation procedures, they may also benefit physicians who are still learning to perform RFA (161).

### Laser ablation

In LTA, the technique starts with inserting one or more introducer needles with a gauge size of 21, keeping a distance of 10 mm between them. Then, 300 μm diameter optical fibers are threaded through the needle sheath. The needle is then removed, leaving at least 5 mm of exposed fiber in contact with the thyroid tissue. A Nd:YAG laser is often used to perform laser safety tests before beginning energy delivery. The entire energy range per illumination falls typically between 1200-1800 J, with an average power per diode of 2-4 W. Real-time creation of a highly echogenic zone because of heating and vaporization is triggered by laser activation. The fiber(s) can be gradually withdrawn by 1-1.5 cm to ensure complete therapy, enabling the administration of additional energy doses. The specific fiber counts, frequency of subsequent applications after retractions, and the overall amount of energy used must be adjusted according to the size and shape of the nodule being treated (28,61,162-165).

### Microwave ablation

An integrally cooled shaft antenna and a generator make up the microwave ablation system. The generator can produce up to 100 W of power while operating at a frequency of 2,450 MHz. The antenna itself is a 16-gauge structure with a 10 cm overall length and a 3 mm active tip diameter. A 1–2 mm skin incision is made to start the treatment so that the antenna can be inserted into the isthmus and then placed inside the target nodule along its longest axis. The moving-shot technique is then applied to treat the target nodule in small units. Usually, the ablation power is changed between 20 W and 50 W. Once the entire nodule appears hyperechoic in the US, the treatment is deemed finished (166-170).

### High-intensity focused ultrasound ablation

The HIFU device consists of various components, including an energy generator, a probe (or transducer), a monitor, and a cooling device. The probe serves a dual function as both an US imaging guidance system and a therapeutic HIFU transducer system. Within the probe, the US imaging component is positioned in the middle to ensure alignment of the treatment focal point with the center of the US image. By placing the probe on the skin, the target nodule becomes visible. The HIFU device then defines treatment and nontreatment areas, with each ablation site measuring approximately 7.3-9 mm by 1.8-5 mm. The operator has the option to manually contour the target on the monitor, viewing it from sagittal and transverse planes. Subsequently, a HIFU pulse lasting 4-8 seconds is administered, followed by a cooling period of 15-50 seconds. The transducer can emit frequencies of 3 MHz with pulses reaching power levels of 125-160 W. Initially, the first pulse is applied at the center of the nodule to assess the formation of hyperechoic white foci. Each pulse generates temperatures between 60 °C and 80 °C, creating an ellipsoidal shape measuring 2 mm in diameter and 9 mm in length. To prevent disruption caused by neck movement during ablation, a laser-based motion detector halts the delivery of the US pulse. Skin cooling is achieved by circulating a liquid at 10°C through a balloon situated within the probe (171-174).

## TECHNICAL RECOMMENDATIONS

**Recommendation 6. Local anesthesia should be used for ultrasound-guided ablation procedures in cases in which patient comorbidities and disposition allow. Mild conscious sedation may be considered to enable monitoring of complications during the procedure, particularly vocal alterations.**

**Recommendation 7a. Hydrodissection is recommended in all cases as it creates a space between the target lesion and vital structures, reducing patient discomfort and minimizing unintended thermal spread and associated complications.**

**Recommendation 7b. If any changes in the patient’s voice are observed during thermal ablation, the procedure should be immediately halted. Rescue hydrodissection using cooled glucose solution (5–10 mL) should then be administered into the tracheoesophageal groove until the voice quality returns to baseline. Corticosteroids may be added to the solution.**

**Recommendation 8a. The transisthmic approach utilizing the moving-shot technique is recommended to minimize accidental thermal damage to critical structures surrounding the target area.**

**Recommendation 8b. For safe and effective ablation during radiofrequency ablation, the moving-shot technique through the transisthmic approach should be employed, and energy should only be delivered when the needle tip is visualized on the ultrasound.**

**Recommendation 9. Continuous monitoring of vital signs for all ablation techniques is not universally necessary. However, if sedation is administered, it is important to follow established guidelines for sedation during procedures in adults.**

### POSTTREATMENT CARE

#### Immediate posttreatment

Following the RFA procedure, clinical and US evaluations should be performed to assess the ablation area, identify any potential early side effects, and confirm that the target lesion has responded well. The treated area appears on US as a somewhat hypoechoic, heterogeneous zone with sporadic hyperechoic areas brought on by tissue dehydration (3) and lacks vascular signals on color Doppler mapping. Contrast-enhanced US, if available, can provide a more accurate assessment of the loss of small vessel signals and a better representation of peripheral areas that have not yet received enough treatment; these areas should be given consideration for subsequent treatment, if safe and necessary (9).

The application of ice packs after therapy can relieve pain, reduce local edema, and promote comfort. For 48 to 72 hours, oral antiinflammatory drugs and common analgesics can be taken together. Antibiotics and corticosteroids should not be used frequently. Before being released, patients should be monitored for at least 60 minutes. Any patient who exhibits hemodynamic irregularities or difficulty breathing, speaking, or eating should be admitted for additional monitoring. It is important to provide detailed postprocedural instructions, including information on symptoms that call for emergency medical attention, such as severe or worsening pain, significant localized swelling and redness, fever, voice changes, difficulty swallowing, or breathing issues (3).

Evaluation of the patient’s voice is crucial for ensuring the safety of the procedure and monitoring complications. Following the completion of treatment, both the patient and the physician subjectively reassess the voice, as done in the preprocedural assessment (143). Any alteration in vocal quality raises concerns about potential injury to the RLN, with the leading causes of paralysis including thermal spread, anesthetic blockade, compression from nodule expansion, or hematoma. In such cases, direct visualization of vocal cord motion through laryngoscopy is recommended (142). Transcutaneous US also allows for laryngeal evaluation and may be more effective in US-guided ablation procedures. However, the ability to adequately visualize the vocal cords can be influenced by factors such as age and sex, while subtle motion abnormalities may be challenging to detect on US (175,176). For patients undergoing bilateral ablation, it is recommended to confirm vocal cord mobility through direct visualization on each side after treatment to avoid the potential complication of bilateral vocal cord paralysis.

## Complications

Due to the RLN’s close proximity to the thyroid and central compartment lymph nodes, the risk of RLN injury during ablation treatments is similar to that of surgery. The absence of studies assessing RLN injury rates utilizing preprocedural and postprocedural laryngoscopy makes it difficult to pinpoint specific RLN damage rates. A systematic review and meta-analysis by Chung and cols. (13) reported a 1.44% overall rate of transient or permanent voice changes after RFA based on subjective voice assessment. The rate of voice changes was higher (7.95%) in a subgroup of 176 patients undergoing RFA for recurrent thyroid cancer located primarily in the central compartment. One strategy proposed to mitigate RLN injury involves the injection of a cold liquid solution into the region suspected of thermal injury when voice changes develop (124). However, it is important to note that previous studies have reported nerve damage due to hypothermia after exposure to very cold solutions (177,178).

Special consideration is necessary for patients considering bilateral thyroid ablation or ablation in the thyroid bed. Close attention should be given to any voice changes, and an examination of the vocal cords is required before proceeding with the contralateral side. Transient edema within the treated nodule is expected after RFA (44); thus, it may be safer to perform bilateral ablations with intervals between them to avoid potential complications, such as delayed nerve paralysis.

Other nerves beyond the RLN may also be thermally injured during thyroid and/or cervical lymph node ablation. These injuries are uncommon and may affect the vagus nerve next to the carotid sheath, the sympathetic chain behind the carotid artery, the brachial plexus in the supraclavicular fossa, and the phrenic nerve in the deep neck musculature (13).

After voice alterations, the second most common complication following RFA is rupture of the nodule (13,128,179). The rupture of the thyroid capsule and nodular growth that result from this complication are related to late bleeding brought on by damage to small vessels (13,180). In this situation, patients frequently report neck pain and edema 2-4 weeks after the treatment, and conservative therapy is usually indicated.

The main factor to consider when choosing RFA over surgery is the prevention of hypothyroidism. In a multicenter study of 1,459 patients with benign nodules treated with RFA, only one patient, who had increased antithyroid antibodies prior to the procedure, was found to have treatment-related hypothyroidism (128). It is also uncommon for hypothyroidism to develop after the removal of an autonomously functioning nodule (181). Due to the concentrated nature of ablation and the frequent unilaterality of the method, postprocedural hypoparathyroidism has not been observed.

Before electrode/needle insertion, it is essential to properly identify the anterior jugular vein or large perithyroid veins on US, as injury to these vessels is the most common cause of hematoma. At the electrode contact site and when ablation is performed on nodules close to the skin, superficial burns may develop. Penetration or ablation close to the trachea may lead to tracheal necrosis and airway impairment (182).

Finally, great care must be taken when thyroid RFA is performed on patients who are pregnant or who have implantable cardioverter-defibrillators (ICDs). Dispersion of electrical current may occur with monopolar electrodes, which are frequently used in thyroid RFA. Concerns arise regarding potential fetal injuries during RFA or interference with the function of the ICD, which poses a life-threatening risk to patients dependent on an ICD for maintaining normal heart rhythm. Although no complications have been reported in these populations, the literature is insufficient. Given the theoretical risk, it would be preferable to utilize bipolar electrodes, which reduce electrical propagation, if RFA is recommended in these patients (159).

## Follow-up

Therapy with RFA is followed by routine clinical, radiological, and biochemical monitoring to evaluate the effectiveness of the treatment and thyroid function.

It is important to note that nodules submitted to ablation may exhibit US characteristics that could be misinterpreted as worrisome, such as a hypoechoic background with internal echogenic foci (152,153). Even though ablated nodules do not exhibit malignant transformation on FNA (183), medical professionals should be aware of these morphological changes. To ensure proper interpretation and prevent unwarranted suspicions and biopsies, it is ideal for the physician who performs the ablation to continue to follow the patient with US evaluations.

For nonfunctioning benign nodules, thyroid function testing and thyroid US should be performed every 3-6 months (2-4). Typically, by 12 months, the expected size reduction has been achieved (184), and thyroid function tests are no longer necessary beyond this period. It is best to use consistent, validated metrics to record symptoms and cosmetic ratings related to nodules. About 5%-24% of treated nodules show regrowth after 3-5 years and may require repeat ablation or surgery (33,185,186); thus, long-term US follow-up is advised in all patients. Decisions regarding repeat ablation versus surgery are based on symptoms, vascularity on Doppler US, remaining nodular volume, and patient satisfaction. In general, cross-sectional imaging (CT and magnetic resonance imaging [MRI]) is not advised unless a nodule with substernal extension is being evaluated.

Due to the potential for more rapid hormonal changes after ablation of an autonomously functioning nodule, thyroid function should be frequently assessed in the first year following the procedure and annually thereafter (2,98,181). As with nonfunctioning nodules, US should be performed at each follow-up. Additionally, repeat scintigraphy should be considered to evaluate the degree of functional response if changes in thyroid function occur. Persistent hyperthyroidism or incomplete response in large nodules with less than a 50% volume reduction may require repeat ablation or alternative therapeutic interventions (33,181).

In thyroid metastasis or recurrent malignant neoplasia, US evaluation during follow-up is focused on volume reduction and intratumoral vascularity for response assessment (49). Serum levels of thyroglobulin (Tg) and thyroglobulin antibodies (TgAbs) should be measured simultaneously. For a more accurate assessment of therapeutic response or identification of newly questionable areas, cross-sectional imaging (CT and MRI) may be an option. Depending on the tumor status, surveillance can be continued after the first year based on the recommended timetable. Depending on the condition of the treated tumors, follow-up with conventional US should be performed at 1 (or 2), 6, and 12 months and then every 6-12 months thereafter (7,49).

Tumor volume, maximal diameter, vascularity, and presence of additional metastatic tumors should all be assessed during the primary tumor’s posttreatment follow-up period. After ablation, serum Tg levels and TgAb levels should also be closely evaluated because, in the majority of patients, serum Tg levels drop quickly (12,49). Since the presence of TgAbs may cause falsely low serum Tg values in immunometric tests (187), serum TgAbs should be evaluated concurrently with serum Tg levels. After surgery, TgAbs may temporarily rise as an apparent immunological reaction, as they may also rise after radioiodine therapy (187).

To assess any remaining tumor or newly formed tumors, thin-slice (<2.5 mm), contrast-enhanced CT imaging can be helpful, especially in the early vascular phase (157). After successful ablation, the recurring tumor’s strong amplification on CT imaging completely vanishes. If a subsequent US or CT reveals the presence of Doppler signals or increased (contrasted) areas of the treated tumor, further ablation may be considered; however, pathological confirmation with FNA or CNB is advised. Notably, contrast-enhanced US has been shown in several trials to characterize the ablation zone better than color Doppler US (114,115).

## MINIMUM REQUIREMENTS FOR TRAINING AND EDUCATION

Thermal thyroid ablation is a technique that requires expertise from different specialties, including endocrinology, interventional radiology, and head and neck surgery. However, the specialty of the physician is less important than the careful selection of patients, knowledge of neck anatomy, and proficiency in cervical US and US-guided procedures. Discussions should emphasize that ablation is one of several treatment options for thyroid nodules, and the optimal approach for each patient should be determined based on multiple factors and decided by both the patient and the physician. Given the diverse backgrounds of physicians performing ablation, it is crucial to ensure the availability of appropriate surgical support in case of complications, such as vocal cord paralysis, airway discomfort, or cervical hematoma. Therefore, an ideal approach involves a collaborative multidisciplinary team to provide comprehensive care for patients undergoing US-guided ablation procedures.

Primarily, physicians intending to incorporate ablation into their practice should possess a thorough understanding of neck diagnosis and interventional US. The success of a US evaluation depends on the training, motivation, and experience of the examiner, as this method is an operator-dependent examination. Initial exposure to US typically occurs during residency and postgraduate training. Depending on their educational background, physicians could require additional official accreditation through programs connected to relevant medical societies, conferences, or continuing education opportunities (188). Expertise is gained by frequent and consistent use of US in clinical practice, not just through formal education or certification (189, 190).

Furthermore, proficiency in thyroid nodule FNA under US guidance is advised. Before undergoing an ablation operation, it is advantageous to be familiar with the transisthmic technique for biopsies. It is advisable to take part in one or more thermal ablation courses, which offer the chance to practice the method on phantoms and observe skilled experts conduct the treatment on patients. Before initiating treatments independently, receiving mentoring from an experienced specialist is essential. According to the literature, specialists who have completed 50 or more RFA ablations have a decreased rate of complications (128). Medical professionals who want to apply thermal ablation ethically in their practice should think of the aforementioned training as a need even though there is no certification process for it. It may be necessary to create institutional standards for carrying out US-guided ablation because there is no set minimum experience requirement to demonstrate competency in such treatments.

It is recognized that the path to acquiring competence in performing the procedure varies based on specialty training experiences and clinical practice. In general, the ideal scenario for incorporating thermal ablation into clinical practice involves a team equipped to provide comprehensive care throughout the diagnostic, intervention, and surveillance process. This includes appropriate follow-up and auditing of treated patients. The attending physician may be a member of a multidisciplinary group that can manage postprocedural clinical, radiological, and biochemical expectations through regular contact. The alignment of all parties involved in the disease management process at every stage of care can be ensured by a multidisciplinary review of cases that are being considered for US-guided procedures (8).

## POSTTREATMENT CARE, FOLLOW-UP, COMPLICATIONS, AND TRAINING RECOMMENDATIONS

**Recommendation 10. It is advisable to meticulously document the progress and, particularly, the complications arising from thermal ablation to provide accurate information to professionals and patients regarding the safety of these procedures.**

**Recommendation 11. Prior advanced training and experience in thyroid and neck ultrasound are indispensable before conducting any ultrasound-guided thermal ablation procedure.**

**Recommendation 12. Proficiency in ultrasound-guided fine-needle aspiration biopsy of thyroid nodules is recommended for physicians performing ultrasound-guided ablation procedures.**

**Recommendation 13. The practitioner should receive specific instructions on the selected ablation technique, with the opportunity to practice on a dedicated model (phantoms) and observe cases performed by other professionals.**

**Recommendation 14. It is vital that initial cases of ablation be supervised by an experienced physician specializing in ultrasound-guided thyroid ablation.**

**Recommendation 15. Physicians conducting ultrasound-guided thyroid ablation but not providing longitudinal patient care should establish communication and facilitate long-term follow-up with a specialized care team responsible for managing thyroid nodular disease.**

**Recommendation 16. Repeat ablation may be considered if the patient remains hyperthyroid after ablation of an autonomous nodule or if the reduction in size of a large benign nodule is less than satisfactory (<50%).**

**Recommendation 17a. After thermal ablation for recurrent metastatic malignancy, it is crucial to monitor and assess the tumor’s volume, vascularity, and locoregional disease status using ultrasound, as well as measure serum thyroglobulin and thyroglobulin antibody levels to evaluate treatment response.**

**Recommendation 17b. In the context of primary malignancy, ultrasound follow-up is necessary to assess the reduction or complete resolution of the malignant tumor, along with long-term evaluation of disease progression and measurement of serum thyroglobulin and thyroglobulin antibody levels to evaluate the response to oncological treatment.**

## FINAL CONSIDERATIONS

This consensus statement was developed by a team of expert physicians with diverse backgrounds, all specialized in thyroid diseases. Interventional radiologists who have completed their training at a SOBRICE-endorsed training center or hold a specialist title in diagnostic imaging with a focus on interventional radiology are recommended to perform the first 10 cases of thyroid ablation under supervision (125). Head and neck surgeons or endocrinologists are advised to undergo US and FNA training courses according to the minimum specifications recommended by their respective medical societies before initiating ablation practice. Subsequently, regular practice in US and FNA should be conducted before attending a thyroid ablation training course. For these specialists, it is also crucial to perform the first 10 cases of thyroid ablation under the supervision of an experienced ablation practitioner (125).

Sponsorship: the present study was developed and supported by the Brazilian Society of Head and Neck Surgery (SBCCP), the Brazilian Society of Endocrinology and Metabolism (SBEM), and the Brazilian Society of Interventional Radiology and Endovascular Surgery (SOBRICE).
